# Perceptions, beliefs, and current practices regarding neonatal skin care and emollient use in eastern Uganda: a qualitative study

**DOI:** 10.1186/s12887-023-04040-y

**Published:** 2023-05-05

**Authors:** Daniel Wenani, Kathy Burgoine, Sarah LA Williams, Milton Musaba, Tewodros Gebremichael, Andrew Clarke, Keona JH Blanks, Ritah Nantale, Jascenti Nawanga, Sarah Kiguli, Mike English, Peter Waiswa, Gary L Darmstadt, Joseph KB Matovu, David Mukunya

**Affiliations:** 1 Department of Community and Public Health, Busitema University, Mbale, Uganda; 2 Neonatal Unit, Department of Paediatrics and Child Health, Mbale Regional Referral Hospital, Mbale, Uganda; 3 Global Programmes Division, Save the Children UK, London, UK; 4 Department of Obstetrics and Gynaecology, Busitema University, Mbale, Uganda; 5 Prematurity Research Center, Department of Pediatrics, Stanford University School of Medicine, Stanford, CA USA; 6 Department of Pediatrics and Child Health, School of Medicine, Makerere University College of Health Sciences, Kampala, Uganda; 7 University of Oxford, Oxford, UK; 8 Department of Health Policy Planning and Management, Makerere University School of Public Health, Kampala, Uganda; 9 Department of Disease Control and Environmental Health, Makerere University School of Public Health, Kampala, Uganda

**Keywords:** Neonatal, Neonate, Skincare, Massage, Emollient, Uganda, Low birthweight, Preterm, Resource-limited setting

## Abstract

**Background:**

The skin is a major route of infection in the neonatal period, especially in low birthweight (LBW) infants. Appropriate and safe neonatal skin care practices are required to reduce this risk. The perceptions and beliefs of mothers and other caregivers towards various neonatal skin care practices in our setting have been documented. Data from Asia suggests that the application of emollient to the skin of LBW infants can promote growth, reduce serious neonatal infections, and potentially reduce mortality. This is the first study to explore the acceptability of emollients and massage as part of neonatal skin care in a low-resource setting in sub-Saharan Africa (SSA) that is representative of the majority of government health facilities in Uganda and many in SSA.

**Objective:**

To explore perceptions, beliefs, and current practices regarding neonatal skin care and emollient use in eastern Uganda.

**Methods:**

We conducted a qualitative study consisting of three focus group discussions (30 participants), eight in-depth interviews with mothers/caregivers of preterm and term neonates and 12 key informant interviews with midwives, doctors and community health workers involved in neonatal care, to explore the perceptions and practices surrounding neonatal skin care and emollient use. Data collected were transcribed and analyzed using thematic content analysis.

**Results:**

Mothers perceived that skin care began in utero. Skincare practices depended on the place of delivery; for deliveries in a health facility the skincare practices were mainly based on the health worker’s advice. Vernix caseosa was often washed off due to its perceived undesirability and was attributed to sexual intercourse in the last trimester. Despite their deleterious attributes found in previous studies, petrolatum-based oils, petrolatum-based jellies and talcum baby powders were the most commonly reported items used in neonatal skin care. In our population, there was high acceptability of emollient therapy use; however, neonatal massage was treated with scepticism as mothers feared damaging the vulnerable neonate. Mothers suggested massage and emollient application be undertaken by health workers, if it becomes an intervention.

**Conclusions:**

In eastern Uganda, the perceptions and beliefs of mothers/caregivers toward neonatal skincare influenced their practices of which some could potentially be beneficial, and others harmful. Emollient use would be easily accepted if adequate sensitisation is conducted and using the gatekeepers such as health workers.

## Background

The skin is a major route of infection in the neonatal period [1–4]. Preterm neonates (born at < 37 weeks completed gestation) are at high risk of infection through the skin because their stratum corneum is not fully developed, thus, barrier function of their skin is compromised. Additionally the skin of preterm infants lacks the naturally protective cutaneous biofilm, vernix [5–7], and is easily injured [4, 8]. Thus, appropriate and safe neonatal skin care practices are required. Neonatal skin care practices include immediate care after birth, bathing, topical application of creams and oils with or without massage [9].

Neonatal skin care practices are known to vary between countries and may even differ within the same country. Studies conducted in South Asia revealed that topical emollient use with massage is a widespread traditional practice and when certain emollients [particularly those containing high concentrations of linoleic acid such as sunflower seed oil] are used, it is an effective strategy for enhancing epidermal barrier function, preventing infections and improving neonatal outcomes [2, 9–11]. Studies in sub-Saharan Africa on perceptions and practices in Ethiopia, Nigeria, Senegal and Tanzania document the use of a variety of emollients, different perceived reasons for their use, and various practices [9, 12]. In Uganda, there are limited data on emollient use. One Ugandan study identified the application of cooking oil for neonatal skin care and was found to be promoted by health workers for maintaining skin health [13, 14]. In central Uganda, the cultural practice of bathing the baby in a herbal mixture called *kyogero* is widely practiced [15]. The perceptions and beliefs of mothers and caregivers towards other various neonatal care practices including early initiation of breast feeding, delayed bathing, skin to skin care, cord care, and thermal protection in our setting have been documented [14–18]. However, no study has explored the use of emollients with or without massage as part of neonatal skin care in Uganda.

There currently remains uncertainty about the effectiveness of emollient therapy and the feasibility of its implementation in sub-Saharan Africa. Before any clinical trials of emollient therapy in Uganda are undertaken, it is essential to understand how emollient therapy and newborn massage align with local beliefs and existing practices. Understanding the beliefs, perceptions and practices around neonatal skincare, exploring the emollients currently in use and assessing the acceptability of beneficial emollients in Uganda will inform the design of future interventional studies in this setting.

## Subjects and methods

### Aim

This study aimed to explore the current practices, beliefs and perceptions towards neonatal skin care and to assess acceptability of emollient use with or without massage in eastern Uganda.

### Study design

This was a descriptive qualitative study to explore perceptions, beliefs, and current practices regarding neonatal skin care and to assess acceptability of emollient use in eastern Uganda.

### Study setting

The study was conducted at Mbale Regional Referral Hospital (MRRH) in Mbale district, eastern Uganda. Uganda is a low-income country with a high neonatal mortality rate (NMR) of 28 deaths per 1000 live births [19].

MRRH has a catchment population of about 4.5 million people in eastern Uganda and acts as the referral facility for 14 districts in and around the Elgon sub-region. The hospital is a government-run facility that offers free health care services. Approximately 10,000 deliveries are performed in MRRH every year. It has a dedicated neonatal unit that admits over 2500 neonates a year, including around 600 low birthweight neonates. MRRH-NNU is recognised nationally as a Centre of Excellence for neonatology, and through implementation of level II neonatal care reduced neonatal mortality from 48 to 21% since 2014 [20]. The current in-patient neonatal mortality is 16% (*personal communication*) and the in-patient mortality of very low birthweight (VLBW, < 1500 g) infants is currently 26% [21].

### Study population and participants’ selection initially

The beliefs, practices and acceptability of emollient were relatively unknown in this population, therefore focus-group discussions (FGDs) with the mothers and caregivers were done initially to stimulate discussion, generate new ideas and promote exploration of the unknown factors. The ideas and perceptions generated from the FGDs were then used to modify the interview guide for the in-depth interview (IDIs) to allow full exploration of these factors.

Three FGDs were conducted with mothers of preterm neonates admitted to the neonatal unit [1], mothers of well term neonates from the postnatal ward [1], and mothers attending the neonatal high-risk clinic at MRRH (Table 1) [1]. To give representative views, the participants were purposively selected to include mothers and caregivers of term and preterm neonates, and various modes of delivery. None of the approached participants declined to participate.

**Table 1:** Participant characteristics by method of data collection

Method	Participant description
**In-depth interviews (IDIs) – n = 8**	• ID1 01- Grandmother of a term neonate in postnatal ward • IDI 02- Grandmother of a preterm neonate in neonatal unit • IDI 03- Maternal aunty of a sick preterm at the high-risk neonatal clinic for review • IDI 04- Young mother with a term neonate at high-risk neonatal clinic for review • IDI 05 and IDI 06- Mothers of preterm neonates at the high-risk neonatal clinic for review • IDI 07 and IDI 08- Mothers of term neonates on the postnatal ward
**Key informant interviews (KIIs)- n = 12**	• Doctors; 2 postgraduate MMed Paediatrics students, 2 intern doctors • Midwives; 3 certificate midwives, 3 diploma midwives • Traditional birth attendant • Community health worker
**Focus group discussions (FGDs) – n = 3, 30 participants**	• FGD1- 10 mothers of preterm neonates delivered by vaginal delivery from the neonatal ward; 1 caregiver of a preterm neonate from the neonatal ward (total n = 11) • FGD2- 11 mothers of term neonates from the postnatal ward, 1 grandmother (total n = 12). 5 were of mothers who delivered by Caesarean section. • FGD3- 7 mothers of preterm neonates from neonatal high-risk clinic

Eight in-depth interviews (IDIs) explored experiences of neonatal skincare practices, perceptions, beliefs and acceptance among mothers and caregivers of neonates. Mothers and caregivers of both term and preterm neonates were purposively selected from the neonatal unit, maternity wards and neonatal high-risk follow-up clinic at MRRH (Table 1).

It was difficult to schedule and recruit enough healthcare workers for a FGD, therefore key-information interviews were used to allow us to collect information from individual experts in neonatal care. Twelve [12] key-informant interviews (KIIs) with health workers [10], traditional birth attendant [1] and community health worker (CHW) [1] were performed (Table 1). We explored their expert opinion on neonatal skincare practices within the hospital and community settings. The traditional birth attendant (TBA) was purposively selected from Wanale sub-county because this sub-county has an organised community referral system and the CHWs from the sub-county have been trained as community health promoters. The CHW from Budaka district was purposively selected because a high volume of referrals to MRRH come from this district.

### Data collection procedures and methods

We conducted face-to-face interviews in a calm and private setting away from any interference having obtained written informed consent from the participants.

We developed separate interview guides for the interviews with the different categories of study participants and topic guides for the different FGDs. This allowed relevant questions to be raised with the different groups. The guides were used flexibly and modified according to the preliminary findings and as the need arose in the study.

We collected data in either a local language (*Lugishu, Luganda*) or English by three trained interviewers; the principal investigator and two Masters of Public Health students. The interviewers were trained on qualitative methodology, principles and practices for one month as a part of their mandatory Qualitative Research methods module in the Master of Public Health curriculum; in addition, the interviewers had a 3-day training about the study protocol and tools used in this study. This training facilitated familiarisation of the study guides on skincare practices and emollient use before the data collection would begin. We ensured consistency among interviewers through the use of interview guides.

Interviews lasted about 20–80 min. A moderator and one note-taker led the FGDs. Participants were briefed on the main purpose of the discussion, and emphasis was placed on inter-participant discussions and confidentiality. We wrote field notes to document impressions while in the field and our own experiences, beliefs, and assumptions. All the IDIs/KIIs/FGDs were audio recorded with permission from the participants. Participants were also shown a video on massage with an emollient (https://www.youtube.com/watch?v=_cwHCclhuGw). Discussions on massage were conducted after the video was shown and responses were again captured through note taking and audio recording.

A professional transcribed and translated interviews and FGDs conducted in *Lugishu or Luganda*. The principal investigator had a good command of Lugishu and proofread the translated transcripts.

The principal investigator was a male master’s student at the time of the study, trained in qualitative methodology. The two research assistants were also masters of public health students, male and female. The principal investigator and one female research assistant belonged to the same ethnic group as most of our mothers and caregivers. This helped understand and interpret some of the descriptions of the various emollients and skincare practices practiced by participants. At the time of this interview, Mbale RRH had a neonatal ward, where we selected some of our participants for FGDs.

### Data analysis

We analysed data that were obtained from the IDIs, KIIs and FGDs using thematic content analysis as described by Braun and Clarke [22]. The six steps as by Braun and Clarke were adapted and included: familiarising with the data obtained, generation of initial codes, search for themes, review of themes, definition and naming of themes, and production of the report.

First, we transcribed the interactions and then read (and re-read) the transcripts and/or listened to the recordings. Initial ideas were noted down, to provide the foundation for the subsequent analysis. After becoming familiar with the data, preliminary codes were identified. The third step in the process was the interpretive analysis of the collated codes. Relevant data extracts were sorted (combined or split). A deeper review of identified themes followed, where initial themes were combined, refined or separated. Themes not related to neonatal skin care were discarded. After reviewing themes, we provided theme names and clear working definitions that captured the essence of each theme in a concise manner. Our analysis was transformed into an interpretable piece of writing by using vivid and compelling extract examples that relate to the themes, research question, and literature.

## Results

In the three focus group discussions with the mothers and caregivers, we found five main thematic areas that described and shaped the current practice and beliefs surrounding neonatal skin care: (1) neonatal skin care starts during pregnancy, (2) early neonatal skin care immediately after delivery, (3) bathing, (4) substances applied to the neonatal skin and (5) substances applied to the umbilical cord stump and cord care. We then explored how these and other factors could enhance acceptability of neonatal emollient use among mothers and caregivers.

### 1 - Neonatal skin care starts during pregnancy

The participants perceived that neonatal skin care began before delivery, and it depended on what pregnant women ate. These beliefs were passed on from elders to pregnant women. Eating vegetables and sunbathing during pregnancy was perceived as beneficial to the skin of the fetus:

*“… while we are pregnant, there is eating of green vegetables which are slippery (‘ndelema’ and ‘Mutele)’ such that the baby’s skin will come out good and healthy after delivery”* (IDI 01, grandmother of a term neonate on the postnatal ward)

*’…for morning’s sunbath when pregnant, I will get vitamin D which helps the yellowish skin of my baby to disappear after delivery’* (IDI 05, a mother of a preterm neonate at high-risk neonatal clinic)

### 2- Early neonatal skin care immediately after delivery

Early neonatal skincare practices immediately after birth varied between mothers who delivered at a health facility and those who gave birth in the community. Practices in the health facilities were influenced by the opinions and advice of the healthcare workers. In contrast, the opinions and recommendations of the CHWs, TBAs and experienced or elderly mothers who have given birth to many children influenced the practices in the community.

Practices related to vernix caseosa removal or preservation, bathing and cord care were most common immediately after delivery and differed between the mothers and the healthcare workers. Mothers believed that vernix caseosa was believed to be a sign of having sexual intercourse during the final trimester, and participants perceived vernix as unclean. Consequently, caregivers would bathe the baby to remove the vernix caseosa despite its biomedical benefits as advocated by healthcare workers.

‘…*that if you give birth to children with these white things on the head, armpits or on the legs, it means you had sex with a man until nine months…’* (IDI 07, mother of a term neonate on the postnatal ward)

*’I heard that it comes when you have sex with a man when the pregnancy is due. I am told those are sperms of a man. I am told that they have nowhere to go because the baby has blocked the way’* (Mother of a term neonate in FGD2)

Conversely, healthcare workers percevied vernix as a protective factor against trauma during in-utero life and infections during the neonatal period, as noted in the quotes below.

*‘When the baby is with that white stuff on the body, it is actually good because that protects the baby. It protects the baby from the womb before delivery and when the baby is born. Some people have a belief that when a baby is born with those white cheesy substances on their body, they think it is ‘sperms’ or it is a sign that the pregnant woman had sex up late during her pregnancy. These beliefs I think they are not true ‘.* (Intern doctor working in the obstetrics and gynaecology department)

*’This natural substance is a protective mechanism for a baby not to get a birth trauma, mostly common in those areas where birth trauma could easily happen on the back, where there is frictions to the bone or to other areas, so the distribution is more in those areas. The substance helps in the prevention on birth trauma by over pressing because it is slippery to help the baby part of the birth canal smoothly.* ' (Diploma midwife working in the maternity ward)

### 3 - Bathing

The timing of bathing varied depending on the place of delivery. Mothers who delivered at the health facilities bathed their babies according to the recommendation and advice of the healthcare workers.

‘*… the mothers with preterm babies take long to bathe their babies as they keep consulting health workers whether they could bathe the babies, so those ones can bathe even after two weeks, they keep wiping until the baby can gain weight.’* (Mother with a preterm neonate in the neonatal ward).

In the health facility setting, the participants understood the importance of delaying bathing, especially for preterm babies, but the same could not be said about practices in the communities. The type of bath given was often characterised by immersion or wiping with a cloth and water everywhere, depending on the status of the neonate (preterm or term babies) and the recommendations of the healthcare workers, as noted by one KII participant.

*‘…from the observations I have made, there are different categories of babies. There are those ones who are delivered in the health facility, many times they are not bathed until the following day because they are kept around for 24 hours. Sometimes they are not bathed in the health facility but are bathed from home after discharge by especially caretakers. Preterm babies are not bathed because the mothers and caretakers think the baby is too small and the fear that the baby might become cold. These small babies are usually only wiped and not bathed by immersing into water until after two weeks when they have gained some weight’* (diploma midwife working in the maternity ward)

*’Our health workers here don’t allow us to bathe the babies while we are here in the health facility on the first day’* (IDI 08, mother of a term neonate on the postnatal ward).

Additionally, the perceived benefits also defined the preferred type of bathing given to neonates by mothers. This was found to be one of the factors influencing the type of bathing, as some mothers argued that they would wipe the baby rather than immerse the baby into the basin. This was due to the belief that immersion bathing would cause discomfort and hypothermia.

Participants who bathed their babies reported using a variety of cleansing agents purchased from the markets, either in liquid or solid form. The choice of these agents depended on the mother’s socioeconomic status or the caretakers. Others, however, preferred herbal-based soaps or other local substances. This was presented as some justification for their use as a remedy for bathing neonates, meaning that their use goes beyond just skin protection. As noted below, two of our participants cited the skin protection benefits during the IDI interview.

*‘The herbal soaps help the baby not to develop scabies, ringworms, and getting rashes.‘* (IDI 04, young mother with a term neonate at high-risk neonatal clinic)

*’We always just use local concoctions like “Bombo” “Kayayana” “Lweza” ‘(*IDI 03, maternal auntie of a preterm in high-risk clinic)

### 4 - Substances applied to the neonatal skin

In Uganda, skincare products are no supplied by the health facility, therefore it is common for mothers and caregivers to bring their own products into the healthcare facility. Participants reported applying various substances on their babies’ skin (Table 2). Substances used included liquid form, jelly-like, oils, and powders. Most mothers reported applying talcum baby powder to the skin of their babies, especially in skin creases, and this was done both in health facilities and in community settings.

**Table 2:** Different types of substances applied onto newborn infant skin

Type/category of substances		Example
Petrolatum-based	Oils	• Cussons^™^ • Johnson and Johnson^™^ baby oil
	Jellies	• Vaseline^™^ • Samona^™^ • Labonita^™^ • Baby Junior^™^ • Sleeping Baby^™^ • Movit^™^ • Labelle^™^ • Baby Mild^™^ • Ballet^™^ • Jelly Tube^™^
Naturally occurring substances		• Coconut oil • Sunflower seed oil • Vegetable cooking oil • Olive oil • Cashew nut oil • Shea butter (*Moo yao*)
Edible fat/magarine		• Blue Band^™^

The jelly-like substances applied to the babies’ skin were both naturally occurring and artificially made. Some of the jelly-like substances which could be equated to fat were butter, cooking fat, or margarine. These were best applied upon melting for easy spreading onto the baby’s skin/body. Most participants reported the use of petrolatum jelly. The common brands on the market include Samona™, Labonita™, Baby Junior™, Sleeping Baby™, Movit™, and Labelle™. Some of these products contained herbs.

Some mothers reported using naturally occurring oils including coconut oil, sunflower seed oil, olive oil, and vegetable cooking oil. Their use was largely reported by mothers of neonates in the neonatal ward at Mbale RRH, where emollient use is promoted. Most other mothers reported the use of petrolatum oils which are artificially made, such as Johnson and Johnson™ and Cussons™.

*‘…they bath their babies and they use these jellies, other use....I see this ones from north they used their oil Moo yao. They used it for their babies and others use cooking oil. But most people use these jellies such as the baby junior™, Movit™ and there is Ballet™.‘* (Certificate midwife working in the maternity ward)

*’In the hospital we tell them how to apply, but if they delivered from the village, they do it by themselves, and it depends on which oil they are using, like in the village they make the oil from the cashew nut tree and then they use it for smearing in the baby’s skin.‘* (Doctor in the obstetrics and gynaecology department*)*

*’Most people use Johnson and Johnson™ baby oil....but other people just apply Vaseline™, Baby junior™, and many others. There is also something that I noticed on the neonatal ward… some mothers apply coconut oil on the skins of their babies’* (diploma midwife working in the maternity ward)

The substances used by participants for skin care were mainly purchased by mothers from markets (ranging from $1–3 for branded products and $0.5-3 for natural oils). Their choice was largely dependent on their social-economic status. Some participants argued that those who couldn’t afford some of the substances often chose to make some of the products locally. Participants who purchased the skin care substances from the markets, reported that they obtained petrolatum-based substances, which are heavily advertised in Uganda.

The mode of application after obtaining the substances was by firstly applying to their fingers and/or palms or cotton, and then spreading and smearing over the entire body. In the preterm neonates, oil tended to be smeared by hand or cottonwool. This was reflected in quotes from our IDIs:

*‘I get some jelly or oil put on the tip of my fingers will, rubbing slowly and not using my palm but the tip of my fingers. At other times I would put the oil onto my palms and then spread and smear onto the entire body of my baby’* (IDI 04, mother of a term neonate)

*‘I put the oil on the cotton then I apply on the entire body. I smear as though I am wiping. It makes the baby’s skin soft and also helps the body not to be inflamed’* (IDI 05, mother of a preterm neonate)

*‘I apply the Movit™ while trying to pull the bones together’* (IDI 03, maternal auntie of preterm neonate).

For those delivered at the health facilities, the applications continue in the community even upon discharge for the next seven days and not until their babies are grown, as described by KII002.

*‘From postnatal ward, after bathing their babies and they then apply these jellies. Others use this oily substance from northern Uganda called Moo yao. I hear others use cooking oil to apply onto their babies’ skins. But most people use other jellies from shops such as the Baby Junior™, Movit™ and Ballet*^*TM*^*’ (KII002)*

The reasons for use of different emollients as reported by participants were embedded in their beliefs about the benefits to the baby, such as softening their skin, smoothening the skin, relaxing the baby, fostering sleep, and protecting their babies’ skin from rashes and infections.

*‘Uhmm... the mothers feel the skin of their babies is dry without applying these oily substances. They say that these substances help to soften and smoothen the skins of their babies and at the same time relaxing the babies to sleep better. Mothers with premature babies we have here on the ward say that these substances help to keep their small babies from infections and rashes’* (KII002)

But contrary to the above justifications, some argued that the skin of the babies is very sensitive and nothing needs to be applied to it, not even water, for at least 24 h after birth, as noted by one key informant (KII006)

*‘ I think the babys skin is very sensitive, but also the babies are still trying to adapt to the new environment, so we don’t normally apply anything not even water for the next 24 hours, yes, not after birth.’* (KII006).

### 5 - Substances applied to the cord and cord care

This study found that no substances were applied to the cord when a mother delivers at the health facility. Participants argued that no substances were applied to the cord while in a health facility setting and that anything applied to it would be upon the recommendation of a health worker.

In home deliveries, we found that various substances of different types were applied to the cord. The rationale for using these substances was to prevent bad smells from infection, and hasten the process of the umbilical cord falling off as recommended by health workers. In most cases, health workers recommend substances like salty lukewarm water, normal saline, and chlorhexidine-based gel (Umbigel™, which is recommended in the Uganda Clinical Guidelines).

*‘So after the baby’s cord is cut, we don’t apply anything, we just clean it, even when we take the mothers to postnatal and even when she goes home we advised them to just clean with saline, that is what we advised them to do, but most of them do their own things, they apply powder, applied what, but we don’t encourage them, those days they used to tell us that we put Umbigel™ but we realised that Umbigel™ give the longer time for the cord to get off’* (KII004)

*’But sometimes when you are in a hospital setting like here, we use normal saline or iodine…. Health workers give us that to make sure the Umbilical cord is clean to avoid infections’* (FGD1 R1)

*’I just wipe using the drip water, to drop on the cord. So that it doesn’t smell, when it started smelling, I was told to put the water’* (IDI 04)

We found that the application of other local substances like powder from the market, cow dung, and lizard droppings were deeply rooted in the cultural practices of some caregivers and mothers. One of the caregivers who was a grandmother reported to have used lizard dung, though currently she reported not using the dung:

*‘Lizard dung was crushed and put on the cord but now if you do, you introduce infections so with my grandchild, I get cotton with drip water and put a drop then I clean the cord until it breaks’.*

A doctor in the obstetrics and gynaecology department also reported that:

*‘When we had not studied yet, and they said, that is off, they would look for the faecal matter of the lizards, now they say when that lizard has defecated, its faeces has two coluors “white and black” (laughs). So they cook it, make sure it’s ready then they apply it on the baby’s cord so that it can dry faster’*

### Acceptability of neonatal emollient use among mothers and caregivers in eastern Uganda

We found that emollient use was widely accepted in the region. Participants said they would accept emollient use if it was beneficial to the babies, like in preventing dry skin of the baby, relaxing the baby and softening the skin of the baby. They also admitted to the fact that it could change practice or be accepted widely and used alongside the traditional ones if there is evidence of its benefits to the baby.

‘*These oils and jellies when applied on the skin of the baby, it moistens and softens the skin while making the baby feel good’* (Mother with a preterm neonate on the neonatal ward)

*‘Uhmm... the mothers feel the skin of their babies is dry without applying these oily substances. They say that these substances help to soften and smoothen the skins of their babies and at the same time relaxing the babies to sleep better. Mothers with premature babies we have here on the ward say that these substances help to keep their small babies from infections and rashes’ (*diploma midwife on maternity ward)

Despite the general agreement among participants regarding emollient use on the skin of their babies, the practice of massaging babies seemed strange. Participants were shown a video of massage and many were worried that massaging could damage their vulnerable neonates.

*‘Aaah, you mean these babies have to be massaged? See massaging the hands, the legs and the back. Won’t that cause the child break because their bones are still weak. I feel these small babies are fragile and delicate”* (mother with a preterm neonate in FGD3)

Among the few participants who admitted to massaging babies were TBAs, and the justification for doing this was straightening the legs of the babies and relaxing them to sleep. Another group of participants who were comfortable with massaging their babies, were the mothers of preterm babies admitted to the neonatal ward. This was one of the recommended practices.

One key informant, a certificate midwife noted that:

Ok, aside from the use of the emollient, I don’t think massage is easily perceived well because they think maybe the baby doesn’t need a massage

Another diploma midwife also noted that:

*“Now I think this thing of massage we have been doing it unknowingly I should be saying to me, because I think I did it on my babies but unknowingly yes, because every attempt to bath the baby and after drying and maybe you are applying a lotion on it. Like myself I used to apply what they called jelly tube, so during that application, automatically, I would massage the baby and pass in the back like stimulating the back, and you go to the limbs as you are massaging, yes.*

When we showed mothers and caregivers a video of how massaging was conducted, they indicated that they would accept it, provided education about the benefits of massage and oil were given and they were told when and how to do it.

*‘I just want to know the number of times I should massage the baby? Do I wipe before applying the oil or you just massage? Do I massage when she is in a pamper or even when she is without? I think when we are taught what to do, how to do it and what time substance to apply we can welcome this idea’* (IDI 05, mother of preterm neonate)’

When asked about who is central in making the decision to massage their babies, the participants stated that the decision whether to massage the baby or not rests with mothers and in some cases, father, grandmothers, relatives, other caretakers or health workers. Some said they do not need permission to massage their babies from anyone. This is reflected in the quotes below:

*‘No one..ahh..aah (laughter), yeah it is my baby...no one is supposed to give me the permission. Aaaaah why would I need permission to massage my baby?’ (*IDI 002, Mother with a 1 day old neonate)

*‘No there is nothing like that, I don’t think there is that need of a union, I think here I am the one taking care of my baby, so I think my decision is good enough’* (certificate midwife)

*’I think no one, because if I think the baby need a massage, I think I will say yes to the massage, though what I have seen in the community is that majority of the grandmothers are the ones who teach and probably they encouraged massaged to be carried out but I think no one would give a permission to do that.‘* (CHW member)

Regarding whether a recommendation to use emollient would change the current neonatal skincare practices, participants suggested that emollient use has the potential to change current neonatal skincare practices if based on evidence of effectiveness.

In most interviews, participants agreed that they would recommend emollient use to their friends, and they advised that emollient use should be rolled out in phases, accompanied by a lot of intensive health education to the public about its benefits. They also suggested engaging the community gatekeepers (CHWs, TBAs, local chairpersons, religious leaders, older women who have given birth to many children) in promoting emollient use. Participants thought the practice of emollient therapy with massage would best be done by health care workers as they felt they could unintentionally damage their babies.

Participants also argued that to facilitate rolling out of the emollient, packaging, affordability, providing samples for mothers and making follow visits to those mothers would be helpful in adopting the idea. Proper packing, as stated by them, would entail precise labelling with clear instructions on the use and benefits. This will also foster its acceptability by mothers of a neonate, as noted in the two IDIs below:

*‘You may have to firstly put labels and then explain about the importance of using this oil if you want other mothers to adopt this nice idea. You need to package your product appropriately’* (IDI 01, grandmother of a term baby on postnatal ward)

*’For other people to quickly adopt this new idea, they may need to see a baby whom this has been applied on and how these oily substances have helped such a baby.’* (IDI 03, maternal auntie of a preterm neonate)

## Discussion

Neonatal skin care practices differ between countries, and to date, much of the published data have come from Asia. Our study is the first study in Uganda to explore the practices, beliefs and perceptions towards neonatal skin care and to assess acceptability of emollient use with or without massage.

**Fig. 1:**
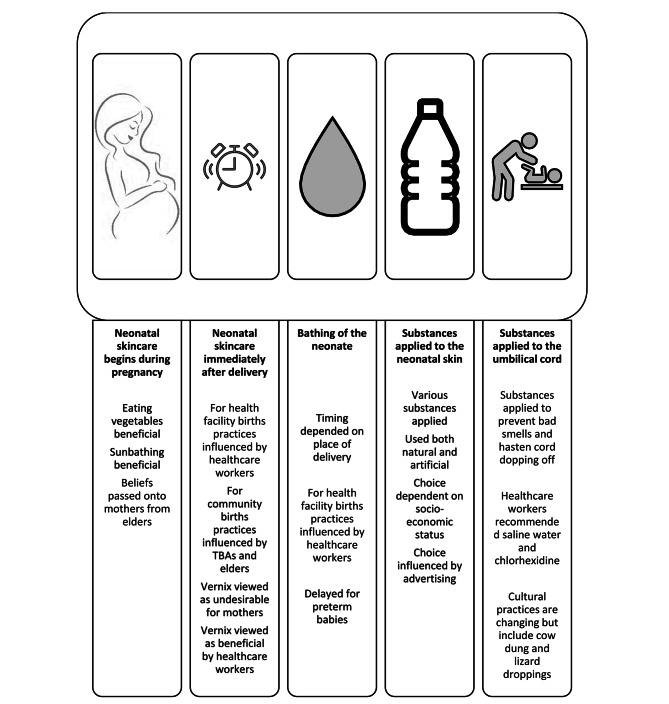
Diagram summarising the five thematic areas and the key factors that influenced that area

Our study revealed the belief that neonatal skin care begins in-utero and described a variety current practices and beliefs surrounding skin care, bathing and cord care, which were beneficial and potentially harmful. These key thematic areas are summarised in Fig. 1.

Practices in the health facilities were influenced by the opinions and advice of the healthcare workers. In contrast, the opinions and recommendations of the CHWs, TBAs and experienced or elderly mothers who have given birth to many children influenced the practices in the community.

In this study, mothers and caregivers had existing perceptions and beliefs which influenced neonatal skincare practices. Our data suggested that these practices were not static but changed with time. These practices appeared to both beneficial and, in some cases, potentially harmful. The perception that neonatal skin care begins in utero was identified. This influenced mothers’ practices during pregnancy, such that they consumed more nutritious food or sought exposure to sunlight to improve their own health and also give birth to healthier babies. Through the use of the health belief model (Rosenstock and Irwin, 1974), this perceived benefit could be useful in the structuring of sensitisation messages to enable mothers to appropriately use emollient therapy in their bid to improve neonatal survival.

Neonatal skincare practices at the time of delivery are influenced by many factors, including the place of delivery (home or health facility), the gestation of the infant at birth (term or preterm), cultural beliefs, perceived benefits and risks, and recommendations from healthcare professionals, especially, in health facilities, and from experienced and influential community members. In mothers who delivered at a facility, most of the practices were shaped by the recommendations of the healthcare workers, for instance bathing the baby is done at least 24 h after delivery as recommended by the midwives and doctors. This could contribute to the adherence towards the acceptance of emollient therapy use in support of future interventional studies and future implementation which could contribute to neonatal survival.

Participants reportedly bathed neonates early in order to remove vernix caseosa, which was perceived as unclean and a sign that a woman had had sexual intercourse late in pregnancy. The vernix caseosa was perceived as sperm. This finding has been reported in other regions in Uganda, Tanzania, Malawi and Senegal [23, 24]. This finding was suggestive of the need for health education on the benefits of vernix in protecting newborns from fluid loss, skin injury and infections both in the community and health facility setting.

The most commonly applied emollients used for neonatal skin care were petrolatum-based oils and jellies. These substances are known to have deleterious effects as demonstrated in prior studies, especially in preterm neonates [25–29]. In addition to emollients, talcum powder (often referred to as baby powder) was commonly applied to the neonates. Talcum powder has recently been banned in high-income countries due to concerns over cancers (mesotheliomas and ovarian cancers); nevertheless, talcum powder is still widely used in eastern Uganda [25, 30].

Some mothers and healthcare workers reported using topical nut oils in our study. Although nut allergy is not a known problem in Uganda, it should be noted that using oils as an emollient creates the potential for topical exposure to allergenic food proteins. Nonetheless, a recent review of topical coconut oil in preterm infants reported no significant adverse effects associated with coconut oil application and another meta-analysis showed that skin care interventions during infancy probably do not change the risk of eczema or the risk of allergic sensitisation to a food allergen by age one to three years [31, 32].

Whereas use of emollient therapy with massage has been widely used as a traditional practice on infants in South Asia, this study highlighted skepticism on the use of massage in neonates, especially preterm babies [33, 34].

In the study of Amare et al., in Nigeria, Ethiopia and Tanzania, it was noted that several factors would influence the acceptability of emollient use, such as cost, social pressure, availability and whether the practice was a traditional norm; in one of the states in Nigeria, massage was done with aggression [12]. In this study, the mothers and caregivers found the use of emollients generally acceptable, but were skeptical about massage. This implies that practice of massaging neonates in sub-Saharan Africa, including Uganda, may be different to the Asian settings. Given that participants were concerned that they could unintentionally damage their babies through massage, careful training and education of the mothers by healthcare workers will be vital to ensure the success of such an intervention.

In this study, mothers and caretakers recommended that emollient use with or without massage would be easily accepted and embraced if the program was implemented and endorsed by healthcare workers. Mandeep et al. developed a theoretical framework of how an intervention, whether a research intervention or a health intervention, can be accepted by the participants/beneficiaries. This framework could be adapted and applied in interventional studies on emollient therapy [35]. This is just like any other public health programming that might require behavioural change messages from the health workers, who are always at the centre of health programs, by engaging the communities on the new approach to skin care.

### Strengths and limitations of the study

We conducted triangulation i.e. using more than one data collection method, allowed increased perspectives and broadened the understanding of the various neonatal skincare practices among mothers of neonates, the perceptions and beliefs around them, and their acceptability. We purposively chose participants to represent a wide range of opinions from different groups with different experiences. However, given the multiple cultural and tribal differences across Uganda, it is possible that these views may not truly represent the views of the wider population in Uganda. There may be other opinions that were not captured but are important for the assessment of the acceptability of emollient use across Uganda. Further qualitative work is therefore needed in Uganda and sub-Saharan Africa to understand the variations between the different regions and tribes. In addition, quantitative evaluation of the number of mothers using emollient therapy and the types, frequency and timing of the emollients being used will be key in understanding the practices in this community.

## Conclusion

Mothers and caregivers had existing perceptions and beliefs which influenced their neonatal skincare practices. The choice of neonatal skincare practice depended greatly on whether the baby was born in a health facility or at home and the practices while in the health facility were largely influenced by the healthcare workers, whereas practices at home were influenced by mothers’ perceptions and beliefs as well as other influential leaders and caregivers, such as grandmothers. Existing practices identified were both beneficial and potentially harmful. We know from other studies that petrolatum based products can damage the neonatal skin [36]. The introduction of emollient therapy would therefore provide an opportunity to replace these harmful practices with a more beneficial practice.

Although not widely practiced, skincare therapy using emollients with proven benefits, such as sunflower seed oil, was generally acceptable. Emollient therapy with massage was treated with scepticism but could potentially be accepted and embraced if community sensitisation and healthcare worker education is done.

## Data Availability

The datasets used and/or analysed during the current study are available from the corresponding author on reasonable request.

## Abbreviations

BSA: Body surface area
CHW: Community health worker
FGD: Focus group discussion
IDI: In-depth interview
KII: Key informant interview
LMIC: Low and middle-income country
LBW: Low birth weight
MRRH: Mbale Regional Referral Hospital
WHO: World Health Organisation
